# Gafchromic EBT‐XD film: Dosimetry characterization in high‐dose, volumetric‐modulated arc therapy

**DOI:** 10.1120/jacmp.v17i6.6281

**Published:** 2016-11-08

**Authors:** Hideharu Miura, Shuichi Ozawa, Fumika Hosono, Naoki Sumida, Toshiya Okazue, Kiyoshi Yamada, Yasushi Nagata

**Affiliations:** ^1^ Hiroshima High‐Precision Radiotherapy Cancer Center Hiroshima Japan; ^2^ Department of Radiation Oncology Institute of Biomedical & Health Science Hiroshima University Hiroshima Japan

**Keywords:** Gafchromic EBT‐XD, radiochromic films, dosimetry, net OD, VMAT

## Abstract

Radiochromic films are important tools for assessing complex dose distributions. Gafchromic EBT‐XD films have been designed for optimal performance in the 40–4,000 cGy dose range. We investigated the dosimetric characteristics of these films, including their dose‐response, postexposure density growth, and dependence on scanner orientation, beam energy, and dose rate with applications to high‐dose volumetric‐modulated arc therapy (VMAT) verification. A 10 MV beam from a TrueBeam STx linear accelerator was used to irradiate the films with doses in the 0–4,000 cGy range. Postexposure coloration was analyzed at postirradiation times ranging from several minutes to 48 h. The films were also irradiated with 6 MV (dose rate (DR): 600 MU/min), 6 MV flattening filter‐free (FFF) (DR: 1,400 MU/ min), and 10 MV FFF (DR: 2,400 MU/min) beams to determine the energy and dose‐rate dependence. For clinical examinations, we compared the dose distribution measured with EBT‐XD films and calculated by the planning system for four VMAT cases. The red channel of the EBT‐XD film exhibited a wider dynamic range than the green and blue channels. Scanner orientation yielded a variation of ∼3% in the net optical density (OD). The difference between the film front and back scan orientations was negligible, with variation of ∼1.3% in the net OD. The net OD increased sharply within the first 6 hrs after irradiation and gradually afterwards. No significant difference was observed for the beam energy and dose rate, with a variation of ∼1.5% in the net OD. The gamma passing rates (at 3%, 3 mm) between the film‐ measured and treatment planning system (TPS)‐calculated dose distributions under a high dose VMAT plan in the absolute dose mode were more than 98.9%.

PACS number(s): 87.56 Fc

## I. INTRODUCTION

Stereotactic radiosurgery (SRS), intensity‐modulated radiation therapy (IMRT), and volumetric‐modulated arc therapy (VMAT) can deliver high doses to targets while minimizing doses to nearby organs at risk (OAR).^1^, ^2^, ^3^ Owing to the complexity of these techniques and patient exposure to high doses, patient‐specific quality assurance (QA) for these treatment techniques is important for confirming the dose distribution, mainly in areas with high‐dose gradients near OARs. Several devices, including matrix detectors^(4)^ portal dosimetry devices^(5)^ and film dosimeters, have been suggested for this purpose. In particular, film dosimetry was initially used as a technique that provides relatively high spatial resolution. Over the past several years, radiographic films, such as EDR2 films, have been widely used in dosimetry measurements despite difficulties associated with the uniform and reproducible development of these films.^(6)^ To avoid this problem, a radiochromic film was introduced as self‐developing dosimetry film. Gafchromic EBT, EBT2, and EBT3 films were released in 2004, 2009, and 2011, respectively, by International Specialty Products (ISP, Wayne, NJ). The properties of these EBT films have been reported in several publications, based on investigations conducted by using several types of photon, electron, and proton beams.^7^, ^8^, ^9^, ^10^, ^11^, ^12^, ^13^, ^14^ Their energy response^7^, ^8^, ^11^ absorption spectra^(9)^ scanning orientation dependence^10^, ^11^ film homogeneity^(12)^ temperature dependence^10^, ^13^ ambient light sensitivity^(10)^ postexposure coloration^10^, ^11^ and variations among different lot numbers^(14)^ were analyzed. The use of EBT3 films is limited to doses below 1000 cGy, owing to the optimality constraint on the film's red channel.^(10)^ Therefore, the verification of high‐dose prescription plans has to use dose scaling.^(15)^

In 2015, ISP introduced a new generation of EBT‐XD model Gafchromic films, specifically designed for optimal performance in the 40–4,000 cGy dose range, making these films best‐suited for applications such as SRS and stereotactic body radiotherapy (SBRT).^(16)^ Before performing patient‐specific QA of radiotherapy treatment plans with the Gafchromic EBT‐XD films, it is important to investigate their dosimetric characteristics. It has been reported that the lateral response artifacts associated with EBT‐XD films are significantly smaller than those associated with EBT3 films.^17^, ^18^

We used standard film dosimetry techniques to study the dosimetric properties of EBT‐XD Gafchromic film, such as its scanning orientation dependence, energy dependence, and postexposure coloration, with applications to high‐dose VMAT verification. For comparison, clinical evaluation and dose‐response measurements were also performed with EBT3 films.

## II. MATERIALS AND METHODS

This study consisted of two parts: 1) an investigation of the film properties, and 2) a clinical evaluation.

### A. EBT‐XD radiochromic film

According to the product specification notes^(16)^ the EBT‐XD film has one, 25μm thick, active layer. The active layer is symmetrically sandwiched between two 125μm thick matte polyester substrates. The active layer and the polyester substrates in the EBT‐XD film are smaller than those in the EBT3 film (active layer: 28μm; polyester substrate: 125μm).

### B. Irradiation procedures

One box of EBT‐XD radiochromic film (Lot #: 01081501) and one box of EBT3 radiochromic film (Lot #: 02261502) were used. The films were handled according to the procedure outlined in the American Association of Physicists in Medicine (AAPM) TG‐55 report.^(19)^ Each film sheet ($20.3\times 25.4\;{\text{cm}}^{2}$) was cut into a 5×4 grid of approximately $4\times 4\;{\text{cm}}^{2}$ pieces that were irradiated to establish calibration curves. The pieces were carefully positioned to ensure the same orientation on the central axis of a tough water phantom (Kyoto Kagaku Co., Ltd, Kyoto, Japan) at a depth of 10 cm. A Varian TrueBeam STx (Varian Medical Systems, Palo Alto, CA) linear accelerator was used to irradiate the films with 10 MV beams with a $3\times 3\;{\text{cm}}^{2}$ field area. The TrueBeam STx can also generate 6 MV flattened X‐ray beams with a 600 MU/min dose rate (DR), as well as 6 and 10 MV flattening filter‐free (FFF) beams with maximum DRs of 1,400 and 2,400 MU/min, respectively. The dose distribution of a 10 MV FFF beam over an area of $3\times 3\;{\text{cm}}^{2}$ appears similar to that of the 10 MV X‐ray beam.^(20)^ We therefore used a $3.0\times 3.0\;{\text{cm}}^{2}$ field area.

### C. Scanning protocol and analysis

An Epson Expression ES‐G11000 (Epson Seiko Corporation, Nagano, Japan) document scanner, with its associated EPSON SCAN v3.49 software, was used. The scanner was set to transmission mode with a 48 bit TIFF image (16 bits per channel), scan resolution of 75 dots per inch (dpi), professional mode, with all available image correction methods turned off. All film pieces were positioned at the same location in the center of the scanning area. Individual channel values were extracted by using IMAGEJ v1.49 software (National Institutes of Health, Bethesda, MD). A $1.0\times 1.0\;{\text{cm}}^{2}$ region of interest (ROI) at the center of each film was selected. The net optical density (OD) was calculated as follows:^(21)^

$$netOD=OD_{\text{exp}}-OD_{\text{unexp}}={\text{log}}_{10}\frac{I_{\text{unexp}}}{I_{\text{exp}}}$$

where $I_{\text{unexp}}$ and $I_{\text{exp}}$ are the readings for unexposed and exposed film pieces, respectively.

### D. Experiments

#### D.1 Dose‐response curve

Fourteen pieces of EBT‐XD and EBT3 films were used to obtain dose calibration curves. One piece was used to obtain the OD of the unirradiated film and 13 pieces were irradiated with normally incident beams with the following nominal doses: 50, 100, 200, 300, 400, 500, 1,000, 1,500, 2,000, 2,500, 3,000, 3,500, and 4,000 cGy. The net OD was obtained for individual channels, and dose‐response curves and their first derivatives were calculated. All irradiated films were scanned 24 hrs after exposure.

#### D.2 Scanner orientation

The dependence of the film orientation on the relative scanning orientation was investigated by scanning the films in the landscape and portrait orientations relative to the scanner. The landscape orientation corresponds to placing a film on the scanner with the film's longer side parallel to the scanner's longer side. The portrait orientation corresponds to placing a film on the scanner with the film's shorter side parallel to the scanner's longer side. To determine possible side dependence, the films were flipped over from front to back for scanning. All irradiated films were scanned 24 hrs after exposure.

#### D.3 Postexposure density growth

Postexposure coloration was analyzed at postirradiation times ranging from several minutes to 48 hrs. The films were irradiated at doses of 500, 1,000, 2,000, 3,000, and 4,000 cGy, with a 10 MV beam.

#### D.4 Energy and dose‐rate dependence

The films were irradiated at doses of 500, 1,000, 2,000, 3,000, and 4,000 cGy, with 6 MV (DR: 600 MU/min), 6 MV FFF (DR: 1,400 MU/min), 10 MV (DR: 600 MU/min), and 10 MV FFF (DR: 2,400 MU/min) beams, to determine any energy and dose rate dependences. All irradiated films were scanned 24 hrs after exposure.

### E. Verification of VMAT QA using EBT‐XD film

**Figure 1:**
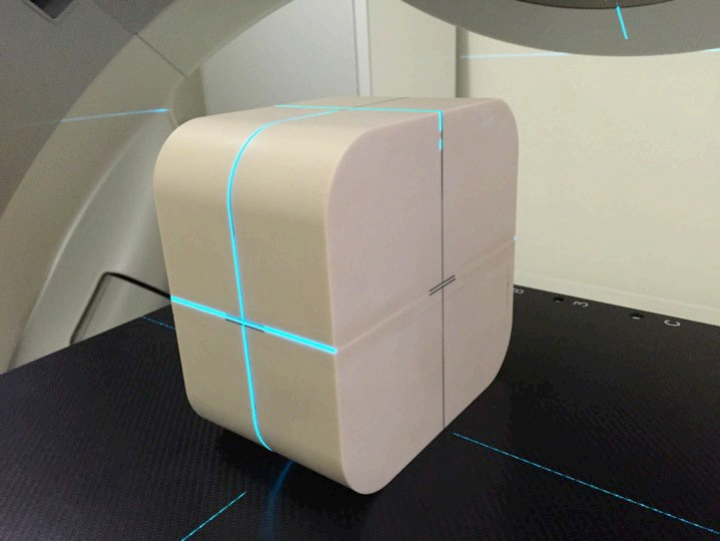
Photograph of the water‐equivalent phantom (height: 20.3 cm, width: 20.3 cm, and length: 12.7 cm) for film measurement. The composite planar dose was measured using a Gafchromic EBT3 and EBT‐XD films, which were inserted in the coronal plane of the phantom.

For clinical evaluation, we used the planar dose distributions of four patients’ VMAT (RapidArc) plans. The VMAT plans were designed with the Eclipse ver. 13.5.35 (algorithm: Acuros XB) (Varian Medical Systems, Palo Alto, CA) treatment planning system (TPS). One fraction of the plan was mapped onto a JUT‐1 phantom (R‐TECH, Inc., Tokyo, Japan), a water‐equivalent cubic phantom made of Tough Water (Fig. 1). The planar dose distributions were exported from the TPS. Central axis doses ranged from 1,266 to 1,704 cGy. The films were placed inside the JUT‐1 phantom in the coronal plane. They were marked with four points, corresponding to the laser for image registration. The plan was delivered on the Varian TrueBeam STx. Film readings (EBT‐XD: red channel, EBT3: red and green channels) were imported into SNC Patient ver. 6.6 (Sun Nuclear Corporation, Melbourne, FL) and compared with the TPS‐calculated dose in the absolute and relative dose modes using a gamma analysis method.^(22)^ The criterion of 3% dose difference (global) and 3 mm distance‐to‐agreement with a 10% threshold was used.

## III. RESULTS

### A. Dose‐response curves

**Figure 2:**
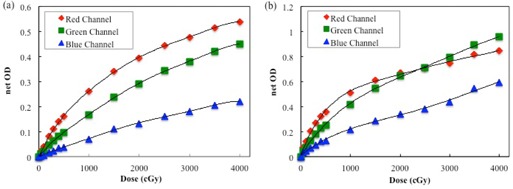
Dose‐response curves of (a) EBT‐XD and (b) EBT3 film using the red, green, and blue channels.

**Figure 3:**
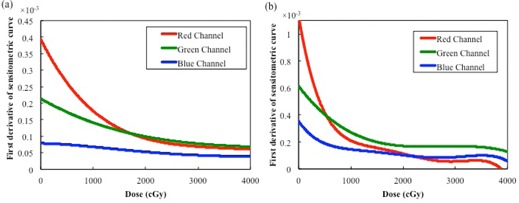
First derivatives of the dose‐response curves for (a) EBT‐XD and (b) EBT3 films.

The net ODs of individual film pieces were analyzed for all three channels. Figure 2 shows the dose‐response curves in the 0–4,000 cGy dose range. The first derivatives of all curves are shown in Fig. 3. The first derivatives for the red channels of EBT‐XD and EBT3 films are the largest up to doses of 1,700 cGy and 500 cGy, respectively, exceeding those for the green and blue channels. The first derivatives for the green channels of EBT‐XD and EBT3 films exceed the first derivatives for the red channels above 1,700 cGy and 500 cGy, respectively.

### B. Scanner orientation

**Figure 4:**
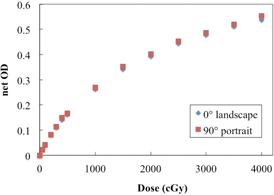
Dose‐response curves for EBT‐XD film when scanned in two different orientations. No significant difference was found with regard to the scanning direction.

**Figure 5:**
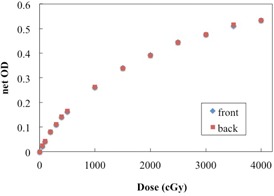
Dose‐responses curve for EBT‐XD film when scanned in side orientation. No significant differences were found with regard to the scanning side.

Film dependence on scanning side and orientation was investigated for the EBT‐XD film. Figure 4 shows the net ODs of the film when scanned in the landscape and portrait orientations. The EBT‐XD film exhibited similar responses in the portrait and landscape orientations. The difference in the net OD was ∼3% (<0.010 diff. in the net OD), for all doses under study. Figure 5 shows the net ODs obtained for both scanning sides, for all doses. The differences between the net ODs of the film when scanned the front and the back were small, with a variation in the net OD of ∼1.3% (<0.010 diff. in the net OD), for all doses under study.

### C. Postexposure density growth

**Figure 6:**
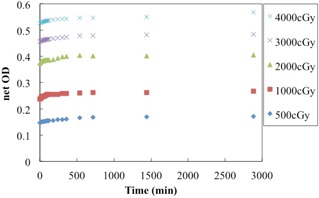
Postirradiation coloration of EBT‐XD film. In all cases, the net OD increased sharply up to 6 hrs after irradiation, and then exhibited a gradual increase.

The net ODs, measured at different times postexposure, are shown in Fig. 6. We compared the net ODs at 30 min, 1 hr, 2 hrs, 3 hrs, 6 hrs, 12 hrs, and 48 hrs after irradiation with the value measured at 24 hrs after irradiation. The net OD increased sharply within the first 6 hrs after irradiation, but only gradually thereafter. The net OD at 6 hrs varied less than 0.010 for all doses under study. Furthermore, the relative change of the net OD was under 0.010 from 24 hrs to 48 hrs after irradiation.

### D. Energy and dose‐rate dependence

**Figure 7:**
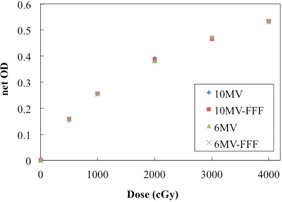
Dose‐response curves for EBT‐XD film for different beam energies and dose rates. No significant difference was found with regard to the beam energy and dose rate.

Figure 7 shows the dependence of the net OD on the beam energy and dose rate. The differences between the net OD values for different energy and dose rates were small, only ∼1.5% (<0.010 diff. in the net OD) for all doses under study.

### E. Verification of VMAT QA using EBT‐XD film

**Figure 8:**
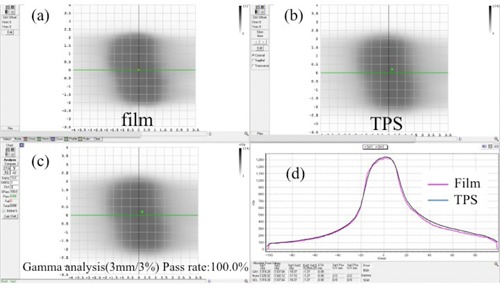
EBT‐XD film (a) measurement data and (b) TPS doses for the VMAT delivery plan. Calculated gamma index map (c) for 3% (global) and 3 mm passing criteria and (d) profiles. Gamma passing rates for the comparison between the TPS‐calculated and film‐measured dose distributions in the absolute dose mode are 100.0%.

Table 1 lists gamma passing rates when comparing TPS‐calculated and film‐measured dose distributions. Figure 8 shows film‐measured and TPS‐calculated dose distributions and the gamma analysis for high‐dose VMAT plan using EBT‐XD film. Gamma passing rates (at 3%, 3 mm) between film‐measured and TPS‐calculated dose distributions under a high‐dose VMAT plan in the absolute dose mode were more than 98.9%. EBT‐XD films can be used for high‐dose film verification in the absolute dose mode, without using the relative dose mode.

**Table 1:** Gamma passing rates (3% and 3 mm) for EBT‐XD and EBT3 films

	*Central Axis Dose (cGy)*	*EBT‐XD Red Channel*		*EBT3 Red Channel*		*EBT3 Green Channel*	
		*Absolute*	*Relative*	*Absolute*	*Relative*	*Absolute*	*Relative*
Plan 1	1,277	100.0%	100.0%	91.6%	99.7%	98.9%	98.7%
Plan 2	1,302	99.1%	99.1%	80.9%	99.9%	96.2%	99.4%
Plan 3	1,704	98.9%	100.0%	100.0%	100.0%	98.9%	99.7%
Plan 4	1,266	98.9%	99.9%	83.8%	98.0%	92.8%	97.8%

Absolute=absolute dose; Relative=relative dose normalized to the isocenter.

## IV. DISCUSSION

In this study, we quantitatively analyzed the effects of various parameters that can potentially influence the measured net OD of Gafchromic EBT‐XD films. For the EBT3 film, the first derivative of the red channel dose‐response curve became small at the dose of ~ 1,000 cGy. The green channel became potentially useful at higher doses. The red channel of the EBT‐XD film exhibited a wide dynamic range, from low to high doses. The blue channel of both film types exhibited a lower response gradient at any dose, owing to the effect of the yellow marker dye. Thus, it seems that the blue channel is less useful for dose measurements than the green and red channels. There have been several reports on triple‐channel dosimetry using the blue channel as an optimal analysis methodology.^(18)^

No significant differences were found between the dose‐response curves of the analyzed EBT‐XD film for different scanning and side orientations. Similar to the EBT3 film, the EBT‐XD film has a symmetric structure. Our results demonstrate weaker dependence than that reported by Andrés et al.^(10)^ for EBT2 films (∼7%–9%) and by Casanova Borca et al.^(11)^ for EBT3 films (∼4.5%). The active particles in the EBT‐XD film are significantly smaller than those in the EBT3 film.^(16)^ Therefore, EBT‐XD films can be scanned with either side facing the light source, and either in the landscape or portrait orientation.

The net OD increased sharply within the first 6 hrs after irradiation and gradually afterwards. The EBT‐XD film exhibited the same behavior as EBT2 and EBT3 films.^10^, ^12^ It should be noted that the difference of 0.010 in the net OD for the EBT‐XD film at 2,000 cGy is equivalent to a variation of 5% in the 100 cGy dose. EBT‐XD films are specifically designed to achieve optimal results in SRS and SBRT, which delivers 600–3,000 cGy per fraction.^(23)^ Thus, the stability of the film at high doses is more important than that at low doses. We recommend a waiting time of over 6 hrs to guarantee adequate stability; otherwise, scanning should be performed with the same waiting time as that used to generate the calibration curve.

We used 6 MV, 6 MV FFF, 10 MV, and 10 MV FFF beams, which are most commonly used in clinical applications. The EBT‐XD film response dependence on energy and dose rates was small in our study. Several groups have reported energy dependence of the responses for EBT film of previous generations. Guerda Massillon et al.^(8)^ reported that the response of EBT3 films only weakly depends on the energy of high‐energy photon beams (6, 15 MV) that are generally used in radiotherapy, and demonstrated a variation under 11% for very low‐energy photons (50 kV). Grams et al.^(17)^ demonstrated minimal energy dependence for EBT‐XD films irradiated with 6–18 MV beams (<0.8%). Further studies are needed to determine the characteristics of EBT‐XD films exposed to low‐energy photon, electron, and proton beams, over a broad range of energies.

In this study, better agreement between the film‐measured and TPS‐calculated dose distributions for high‐dose VMAT plans was found with the EBT‐XD films than with the EBT3 film. This may be attributed to the facts that the slopes of the response function for the EBT‐XD film have a wider dynamic range than those for the EBT3 film at doses > 1,000 cGy and that the EBT‐XD film has wider dynamic range than the EBT3 film.

## V. CONCLUSIONS

The red channel of the EBT‐XD film exhibited dynamic range, from low to high doses. Scanner orientation yielded a variation of ∼3% in the net OD. The difference between the film front and back scan orientations was negligible. No significant difference was observed across different energies and dose rates. The net OD increased sharply within the first 6 hrs after irradiation and gradually afterwards. The EBT‐XD film exhibited a weak dependence on clinically relevant energy and dose‐rate values. Good agreement was found between the EBT‐XD film‐measured and TPS‐calculated dose distributions for high‐dose VMAT plans.

## COPYRIGHT

This work is licensed under a Creative Commons Attribution 3.0 Unported License.
